# Effects of Chronic Sleep Deprivation on the Extracellular Signal-Regulated Kinase Pathway in the Temporomandibular Joint of Rats

**DOI:** 10.1371/journal.pone.0107544

**Published:** 2014-09-16

**Authors:** Chuan Ma, Gaoyi Wu, Zhaoling Wang, Peihuan Wang, Longmei Wu, Guoxiong Zhu, Huaqiang Zhao

**Affiliations:** 1 Department of Stomatology, Jinan Military General Hospital, Jinan City, Shandong Province, China; 2 College of Stomatology, Shandong University, Jinan City, Shandong Province, China; 3 Shandong Provincial Key Laboratory of Oral Biomedicine, Jinan City, Shandong Province, China; 4 cardiovascular medicine, He Bei medical University, Shijiazhuang City, Hebei Province, China; Northwestern University Feinberg School of Medicine, United States of America

## Abstract

**Objectives:**

To examine the possible involvement and regulatory mechanisms of extracellular signal-regulated kinase (ERK) pathway in the temporomandibular joint (TMJ) of rats subjected to chronic sleep deprivation (CSD).

**Methods:**

Rats were subjected to CSD using the modified multiple platform method (MMPM). The serum levels of corticosterone (CORT) and adrenocorticotropic hormone (ACTH) were tested and histomorphology and ultrastructure of the TMJ were observed. The ERK and phospho-ERK (p-ERK) expression levels were detected by Western blot analysis, and the MMP-1, MMP-3, and MMP-13 expression levels were detected by real-time quantitative polymerase chain reaction (PCR) and Western blotting.

**Results:**

The elevated serum CORT and ACTH levels confirmed that the rats were under CSD stress. Hematoxylin and eosin (HE) staining and scanning electron microscopy (SEM) showed pathological alterations in the TMJ following CSD; furthermore, the p-ERK was activated and the mRNA and protein expression levels of MMP-1, MMP-3, and MMP-13 were upregulated after CSD. In the rats administered with the selective ERK inhibitor U0126, decreased tissue destruction was observed. Phospho-ERK activation was visibly blocked and the MMP-1, MMP-3, and MMP-13 mRNA and protein levels were lower than the corresponding levels in the CSD without U0126 group.

**Conclusion:**

These findings indicate that CSD activates the ERK pathway and upregulates the MMP-1, MMP-3, and MMP-13 mRNA and protein levels in the TMJ of rats. Thus, CSD induces ERK pathway activation and causes pathological alterations in the TMJ. ERK may be associated with TMJ destruction by promoting the expression of MMPs.

## Introduction

The temporomandibular joint (TMJ) is a specialized synovial joint essential for the function of the mammalian jaw, and it plays an important role in craniofacial growth and function. Temporomandibular disorder (TMD) is a functional disorder of the TMJ and has been reported to affect an estimated 9%–15% of the adult population in North America [1]. Although the psychological factoris considered to be an etiology of TMD, only a few studies have focused on whether psychological factors could lead to pathological changes in the TMJ or TMD [2]. Another study has reported that psychosocial factors are important in the etiology and maintenance of TMD [2]. Studies have consistently shown that the majority of patients with TMD report poor sleep quality and that the subjective ratings of poor sleep are associated with increased severity of clinical pain and psychological distress [4]–[6]. Many studies of sleep disturbance in TMD are epidemiological reports, clinical case studies, or questionnaire surveys [7], [8]. However, few well-controlled experiments have been carried out on sleep disorders in TMD because it is difficult to establish a research model of sleep disturbance on TMD and involves many factors that are complicated to index and quantify.

ERK belongs to the mitogen-activated protein kinase (MAPKs) family. MAPKs are a family of structurally related serine/threonine kinases involved in cellular events such as growth, differentiation, and stress responses [3]. ERK is activated by MAPK kinase (MEK) as part of the MAPK pathway [10], [11]. Activated ERK can translocate to the nucleus and activate transduction factors by phosphorylation, thus altering the expression of specific genes. Several studies have demonstrated that these cascades are vital to the synthesis of many catabolic factors responsible for inducing synovitis and cartilage destruction [10], [12]. ERK has been found to be a key factor in the induction of many matrix metalloproteinase (MMP) subtypes (e.g., MMP-1, MMP-3, and MMP-13) [13]–[15].

Upregulation of MMPs is widely known to be closely related to the occurrence and development of TMD [16]–[18]. ERK is highly expressed in large joints of patients with rheumatoid arthritis (RA) and osteoarthritis (OA) [12], [19]. It is seldom reported that the ERK pathway is activated in the synovial membrane or articular cartilage in TMD patients and in experiments [4].

Therefore, the present study aimed to assess the histomorphology and ultrastructure of the TMJ and to examine the possible involvement of ERK and its regulatory mechanisms after CSD in a rat sleep deprivation model.

## Materials and Methods

### Ethics statement

Prior approval from the Animal Care and Use Committee of Jinan Military General Hospital was obtained in accordance with international guidelines for care in animal research. The protocol (Permit Number: IACUC-2013-001) was approved by the Committee on the Ethics of Animal Experiments of Jinan Military General Hospital. All surgery was performed under sodium pentobarbital anesthesia, and all efforts were made to minimize rat suffering.

### Experimental design

Two hundred and seventy male 8-week-old Wistar rats (weighing 200–220 g) were purchased from the Laboratory Animal Center of Shandong University (Jinan, China). The animals were housed in 80 cm×45 cm×40 cm cages in a temperature-controlled room at 24°C under a 12-hour light-dark cycle and given free access to food and water. The animals were acclimated to laboratory conditions for one week, and adapted to the CSD for 30 min per day for five consecutive days before the start of the experiment.

The rats were then randomly divided into three groups (n = 90 per group): the control (CON) group, chronic sleep deprivation (CSD) group, and the chronic sleep deprivation with U0126 injection group (U0126 group). The three groups were equally divided into three subgroups (n = 30 each) according to the observation time points (7, 14, and 21 days). The CSD and U0126 rats were placed on small platforms during the procedure, as described in a subsequent section in this paper. The rats in the U0126 group were given intra-articular injections of 5 µg U0126 (Promega Corporation, USA) dissolved in 50 µL of saline into the TMJs twice a week during the experimental period. The CON rats were placed on a grid under the same conditions.

After 7, 14, and 21 days of sleep deprivation, blood samples were obtained from the cardiac ventricles of CSD and CON group rats between 09∶00–12∶00 o’clock under anesthesia by intra-peritoneal injections of pentobarbital sodium (50 mg/kg body weight). The serum was separated by centrifugation (3,000×*g* for 10 min at 4°C) and stored immediately at −80°C for the hormone tests described below. All the animals were sacrificed by an overdose of pentobarbital sodium and their bilateral TMJs were removed. The TMJs were dissected, and 10 right joints were randomly selected from each group for hematoxylin and eosin (HE) staining, and 10 left joints were randomly selected for ultrastructural observations using scanning electron microscopy (SEM). Additionally, 40 joints were selected from the remaining 20 rats in each group to determine the expression of ERK and MMP by Western blotting and real-time quantitative polymerase chain reaction (PCR). The TMJ specimens were dissected and flash frozen with liquid nitrogen. Two pieces of condylar cartilage from each rat were regarded as one sample to ensure that enough protein or RNA was available for the analysis.

### Animal model for CSD

**Figure 1:**
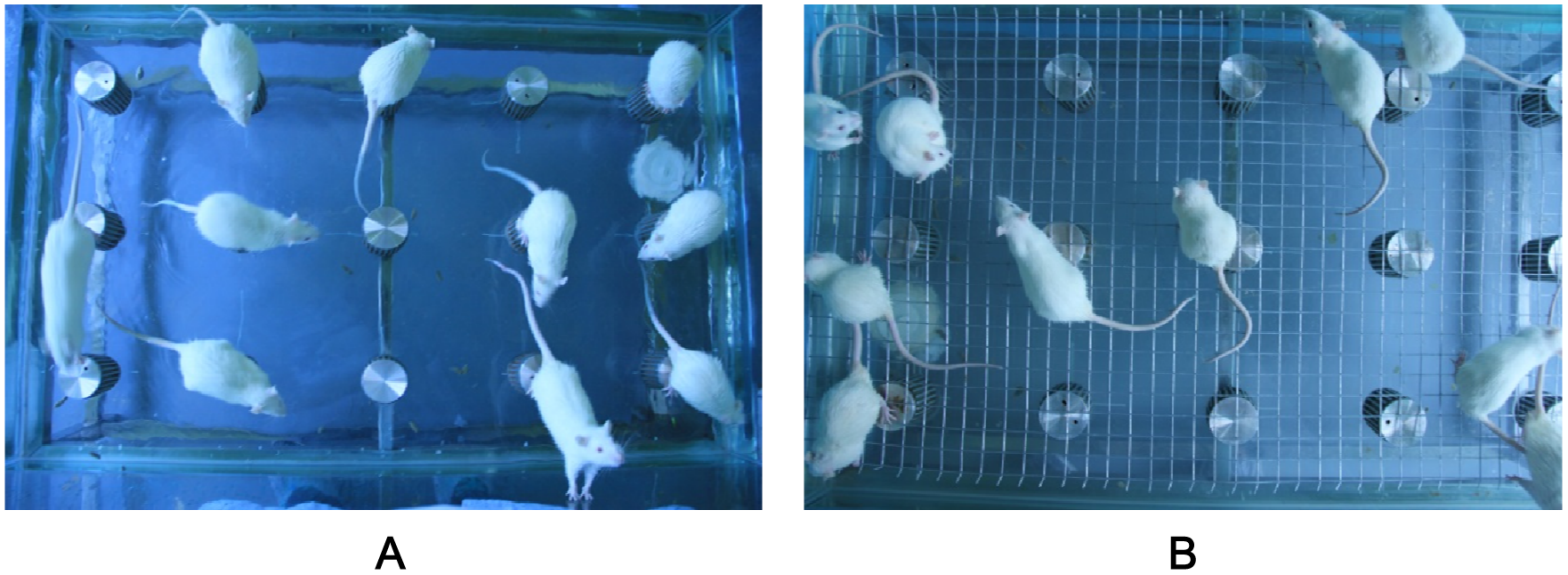
The modified multiple platform method. Sleep deprivation was achieved by depriving the rats of paradoxical sleep. (A) Photos of circular narrow platforms with rats inside. (B) Photos of a grid floor with rats inside. The tank is filled with water up to approximately 1 cm from the surface of the platform or grid.

The modified multiple platform method (MMPM) was selected to induce CSD in this study [5]. As shown in Figure 1, the rats were placed inside a tiled glass water tank containing 15 narrow circular platforms (6.5 cm in diameter) or a grid floor. The grid was made of stainless steel with the rods set 2 cm apart, and it was used to establish an environmental control group for sleep deprivation. The tank is filled with water until approximately 1 cm from the platforms or grid’s surface. The rats that were placed on the grid could lie down without falling into the water, albeit their tails may touch the water. The rats were allowed to move around freely inside the tank by jumping from one platform to another. When they reached the paradoxical phase of sleep, the rats were awakened when their faces would touch the water as a result of the muscle atonia. Thus, sleep deprivation was achieved by depriving the rats of paradoxical sleep. Throughout the study, the temperature of the experimental room was controlled at 23±1°C and a 12∶12-h light-dark cycle was used (lights on at 07∶00 h and off at 19∶00 h). Food and water were provided ad libitum by placing chow pellets and water bottles on a grid located at the top of the tank. The water in the tank was changed daily throughout the CSD period.

After the adaptation period, the rats were placed in the MMPM and subjected to sleep deprivation for 18 h (starting at 16∶00 o’clock) every day for 21 consecutive days. After each 18-h sleep deprivation period, the animals were allowed to sleep in their individual home cages for 6 h (beginning at 10∶00 o’clock).

### Serum assay

The serum concentrations of CORT and ACTH were measured by radioimmunoassay using an Access Immunoassay System (Beckman Coulter, USA) according to the manufacturer’s protocols.

### Histological staining

The condyle and articular disk were fixed in 10% buffered paraformaldehyde, decalcified with 10% ethylene diamine tetraacetic acid (EDTA) at 4°C for 4 weeks and embedded in paraffin wax. The serial 5-µm sections were cut along the sagittal plane and stained with HE. The central portions of each stained section were examined under a light microscope (DM 2500, Leica, Germany). Image acquisition was performed using the Leica DFC490 system (Leica, Germany).

### Preparation of scanning electron microscopic samples

The condyle was fixed with 25 g/L glutaraldehyde for 48 h at 4°C, washed with phosphate buffer (0.1 mol/L), and fixed in osmic acid (10 mol/L). The samples were dried at the critical point, vacuum sprayed, and observed under a scanning electron microscope (JEM-100SX, JEOL Company, Japan).

### Western blotting

TMJ tissues were mixed with cold lysis buffer (Beyotime, China) and 1∶100 volume of phenylmethanesulfonyl fluoride and homogenized in a gentle MACSTM Dissociator (Miltenyl Biotec, Germany). The samples were centrifuged at 14,000×*g* for 5 min to remove debris. The protein concentrations were measured using a BCA kit (Beyotime, China), and then 50-µg protein samples were separated by sodium dodecyl sulfate-polyacrylamide gel electrophoresis (SDS-PAGE) and transferred onto a polyvinylidene fluoride (PVDF) membrane (Millipore, USA). The PVDF sheet was blocked with 5% non-fat dried milk in Tris-buffered saline containing 0.1% Tween-20 at room temperature for 1 h, and incubated with primary rabbit polyclonal antibodies against rat antigens. The following antibodies were used to detect proteins: rabbit anti-MMP-1 polyclonal antibody (1∶400 dilution; lot no. bs-4597R, Bioss, China), anti-MMP-3 (1∶500 dilution; lot no. BS1238, Bioworld, China), anti-MMP-13 polyclonal antibody (1∶500 dilution; lot no. BS-0575R, Bioss), anti-total ERK (1∶3,000 dilution; lot no. #1695, Cell Signaling Technology, USA), and anti-p-ERK (1∶1,000 dilution; lot no. #4370, Cell Signaling Technology). The blots were developed using a horseradish peroxidase-conjugated secondary antibody (Beyotime, China) and enhanced chemiluminescence using an ECL chemiluminescence kit (Beyotime). The blots were exposed to autoradiographic film for 1–2 min for detection.

### Reverse transcription and real-time quantitative polymerase chain reaction (RT-qPCR) analysis

Total RNA was extracted using TRIzol reagent (Invitrogen, USA) according to the manufacturer’s instructions. The TMJ tissue was ground into powder in liquid nitrogen using a gentleMACS Dissociator (Miltenyl Biotech, Germany), and reverse transcription and RT-qPCR were carried out using an Ultra SYBR Two Step RT-qPCR Kit (with ROX; CW Biotech, China) according to the manufacturer’s instruction. RT-qPCR was carried out in an Eppendorf Realplex 4 (Eppendorf AG, Germany) with the following settings: 10 min of pre-incubation at 95°C followed by 40 cycles of 20 s at 95°C and 60 s at 55°C. The 25-µl reaction volume contained 2× UltraSYBR mixture (with ROX), forward and reverse primers (10 µm), and template cDNA. Melting curve analysis was carried out using the default program. After each reaction, the cycle threshold (Ct) was recorded when the amplification curve reflected the exponential kinetic measurements. The 2^−ΔΔCt^ method was adopted with GAPDH as the reference gene [6].

The primers for rat MMP-1 (forward: 5′-CTCCCTTGGACTCACTCATTCTA-3′, reverse: 5′-AGAACATCACCTCTCCCCTAAAC-3′), MMP-3 (forward: 5′-ATGATGAACGATGGACAGATGA-3′, reverse: 5′-CATTGGCTGAGTGAAAGAGACC-3′), MMP-13 (forward: 5′-GCGGTTCACTTTGAGGACAC-3′, reverse: 5′-TATGAGGCGGGGATAGTCTTT-3′), and GAPDH (forward: 5′-CAGTGCCAGCCTCGTCTCAT-3′, reverse: 5′-AGGGGCCATCCACAGTCTTC-3′) were designed with Primer Premier Version 5.0 software and their efficiency was confirmed by sequencing their conventional PCR products.

### Statistical analysis

All data were expressed as means ± standard error. Experimental data were analyzed by one-way analysis of variance (ANOVA). Relative indices were analyzed using SPPS version 13.0 software (SPSS, USA). The Student-Newman-Keuls q test was further used to calculate any differences between the groups. A *P*-value of less than 0.05 was considered statistically significant.

## Results

### Increased concentration of serum CORT and ACTH

To verify that the experimental rats were under CSD stress, we analyzed the serum CORT and ACTH levels. As shown in Table 1, the CORT concentrations after 7, 14, and 21 days of sleep deprivation were significantly higher in the CSD group than in the CON group (*P*<0.05). Similarly, the serum ACTH concentrations after 7, 14, and 21 days of sleep deprivation were significantly higher in the CSD group than in the CON group (*P*<0.5, indicating that the rats in the CSD group were under sleep deprivation stress.

**Table 1:** CORT and ACTH levels in serum.

	CORT (ng/ml)		ACTH (pg/ml)	
	CON	CSD	CON	CSD
7d	5.26±0.84	7.94±1.39*	124.37±15.97	197.64±23.64*
14d	5.63±0.74	9.43±0.89*	130.66±13.23	213.21±17.90*
21d	5.60±1.04	9.63±1.70*	135.29±16.71	206.84±24.52*

CORT, cortisol; ACTH, adrenocorticotropic hormone; CON, control; CSD, chronic sleep deprivation; d, days.

*P<0.05, significantly different from the control group. Data are represented as the M ± SD of n = 10. M, mean; SD, standard deviation; n, sample size.

### Histological observations

**Figure 2:**
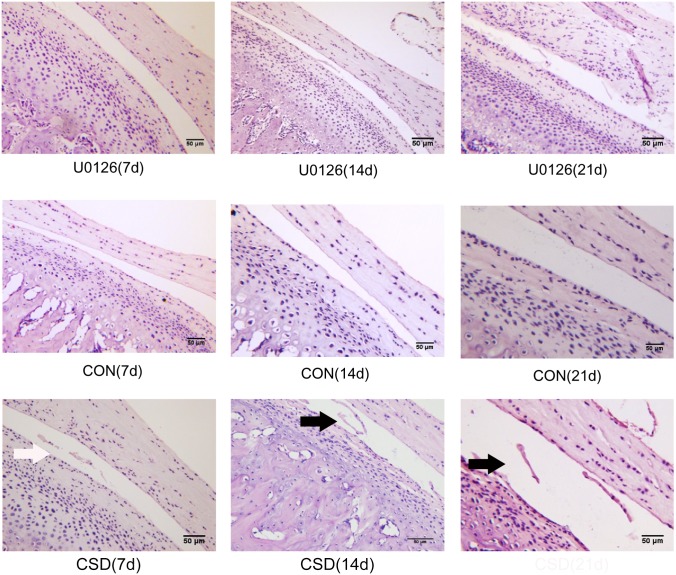
Sagittal section of the rat temporomandibular joint (TMJ) stained with hematoxylin and eosin. The intermediate zone of the articular disk and corresponding condylar cartilage were selected for histological observation. Upper panel: Central condyles of the U0126 injection group rats at 7, 14, and 21 days of sleep deprivation. Middle panel: Central condyles of the control group rats at 7, 14, and 21 days. Lower panel: Central condyles of the CSD group rats at 7, 14, and 21 days of sleep deprivation (original magnification: ×200, scale bar = 50 µm).

We selected the intermediate zone of the articular disk and corresponding condylar cartilage for histological observation. As shown in Figure 2, the condyles of the CON group displayed characteristic zonal cellular arrangements with distinct regions in the articular cartilage. No obvious histological changes were found in the condylar cartilages of the CON group. In the CSD group, the fibrous articular surfaces of the condylar cartilages became visibly tougher (white arrow) at 7 days of sleep deprivation (7/10 rats), and a debonding fibrous layer (black arrow) appeared in the majority of samples at 14 (8/10 rats) and 21 days of sleep deprivation (8/10 rats).

Although histopathological changes such as tough fibrous articular surfaces and a fraction of distorted collagen fibers could be observed in the U0126 group, no debonding fibrous layer was observed and fewer TMJ samples showed histological changes (4, 6, and 5 of 10 rats at 7, 14, and 21 days of CSD, respectively) compared with the number of samples in CSD group. Therefore, these findings confirmed that sleep deprivation may cause pathological alterations in rat TMJ, which could be reversed by ERK inhibitor.

### Ultrastructure analysis

**Figure 3:**
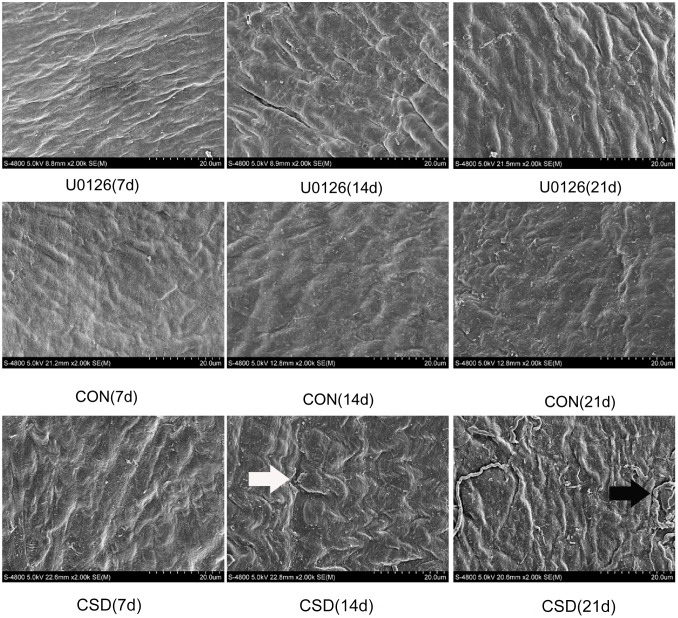
Ultrastructure of the condyle visualized by scanning electron microscopy (SEM). SEM was used to observe the subtle ultrastructural alterations of the TMJ. Upper panel: Condylar fibrous articular surfaces of the U0126 rats at 7, 14, and 21 days of sleep deprivation. Middle panel: Condylar fibrous articular surfaces of the control group at 7, 14, and 21 days. Lower panel: Condylar fibrous articular surfaces of the CSD group at 7, 14, and 21 days of sleep deprivation (original magnification: ×2,500).

To observe the subtle ultrastructural alterations of the TMJ, we used SEM to examine the condyle in the three groups after 7, 14, and 21 days of sleep deprivation. As shown in Figure 3, smooth condylar fibrous articular surfaces and compact bundles of collagen fibers were observed in all the CON subgroups, whereas the CSD rats at 7 days showed more apparent ripples of collagen fiber bundles. The surfaces of the fibrous chord appeared rough and the uniform distribution of collagen fibers (white arrow) was disturbed in the CSD group at 14 days. In the CSD rats at 21 days, the waves of the collagen fibers became wider and cracks (black arrow) appeared on the surface of the condylar collagen.

Although similar ultrastructural observations were found in the U0126 group, such as twisted bundles of collagen fiber and cracks in a fraction of fibrous surfaces, the width of the collagen fiber waves was lower and the worn strips on the condylar surface showed relatively lower severity than in the CSD group.

### Activation of p-ERK and upregulation of MMP expression in rat condylar cartilage

**Figure 4:**
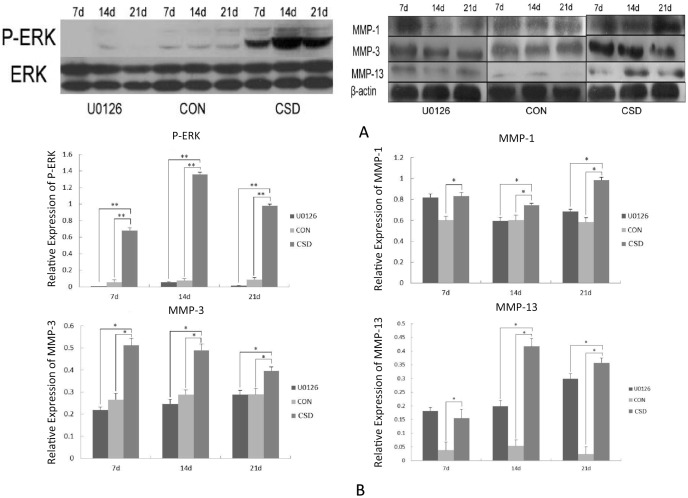
Expression levels of p-ERK, ERK, MMP-1, MMP-3, and MMP-13 in the condyles. Western blot technique was used to examine the possible mechanism by which pathological alterations occur in the TMJ. (A) Comparison of the p-ERK, ERK, MMP-1, MMP-3, and MMP-13 protein levels in the different groups as determined by Western blot (WB). (B) Mean relative protein levels of p-ERK, ERK, MMP-1, MMP-3, and MMP-13 in different groups (n = 10 per group). Bars represent the mean and SD of each group. CON, control; CSD, chronic sleep deprivation; d, day. **P<0.01, *P<0.05.

We attempted to investigate the possible mechanism by which pathological alterations occur in the TMJ; to this end, we measured the expression levels of ERK and MMPs in the mandibular condylar cartilages. As shown in Figure 4, after 7, 14, and 21 days of sleep deprivation, the p-ERK was significantly activated in the CSD group (*P*<0.01) as compared with that in the control group, in which p-ERK was mostly not activated. In terms of the total ERK, there were no obvious changes in both the CSD and control groups. Similarly, after sleep deprivation, both protein and mRNA expressions of MMP-1, MMP-3, and MMP-13 were upregulated in the CSD group compared with the control group. As shown in Figure 4, with increasing duration of sleep deprivation, the MMP-1, MMP-3, and MMP-13 protein levels increased significantly in all the sleep-restricted groups (*P*<0.05) as compared with the control group. RT-qPCR revealed the expression of MMP-1, MMP-3, and MMP-13 mRNA (Tables 2), and the expression levels were significantly higher in all the sleep-restricted groups (*P*<0.05) as compared with the control group.

**Table 2:** MMP-1, 3, and 13 mRNA relative levels in the condylar cartilage.

	CON	CSD	U0126
	MMP-1	MMP-3	MMP-13	MMP-1	MMP-3	MMP-13	MMP-1	MMP-3	MMP-13
7d	0.73±0.14	1.13±0.24	0.72±0.43	1.06±0.28*	3.09±0.44*	3.45±0.54*	1.12±0.32	1.25±0.15#	3.03±0.41
14d	0.82±0.21	1.38±0.41	0.88±0.29	1.87±0.42*	2.56±0.39*	5.32±0.61*	0.94±0.29#	1.43±0.32#	3.72±0.38#
21d	0.87±0.19	1.22±0.36	0.61±0.36	2.39±0.35*	2.31±0.47*	4.69±0.65*	0.91±0.37#	1.08±0.29#	3.65±0.55#

The 2^−ΔΔCt^ method was adopted with GAPDH as the reference gene.

CON, control; CSD, chronic sleep deprivation; chronic sleep deprivation with U0126 injection; d, days.

*P<0.05, significantly different from the control group;

# *P*<0.05, significantly different from the CSD group. Data are represented as the M ± s of n = 10. M, mean; SD, standard deviation; n, sample size.

These results showed that p-ERK was activated and the MMP expression was upregulated in the condylar cartilage as a result of sleep deprivation.

### ERK inhibitor downregulates the expression levels of MMP genes

Next, in order to investigate whether activated p-ERK was involved in the upregulation of MMPs, the articular cavities of one group of rats were injected with the specific ERK inhibitor U0126. The p-ERK, total ERK, MMP-1, MMP-3, and MMP-13 expression levels in the condylar cartilages were then measured. As shown in Figure 4, the p-ERK level in TMJs injected with U0126 showed an obvious decrease (*P*<0.01) as compared with the CSD subgroups that did not receive U0126. The total ERK level, however, showed no obvious changes between the groups that did and did not receive U0126. Both protein (Figure 4) and mRNA (Table 2) expression levels of MMP-3 in all the U0126-treated groups were significantly downregulated (*P*<0.05) as compared with the groups that did not receive U0126. The MMP-1 and MMP-13 protein (Figure 4) and mRNA (Table 2) expression levels in the U0126 group at 14 and 21 days of sleep deprivation were downregulated (*P*<0.05) as compared with the corresponding levels in the CSD group. As mentioned above, the TMJs that received U0126 injection showed fewer histological alterations in the joint cartilage as compared with those that did not receive U0126.

These findings suggest that the activated ERK pathway was involved in the regulation of the MMP-1, MMP-3, and MMP-13 expression.

## Discussion

The present study demonstrated that CSD leads to pathological changes in the TMJ and upregulates the expression and secretion of MMP-1, MMP-3, and MMP-13 by activation of the ERK signaling pathway in TMJ condyles of rats. These findings provide important new evidence indicating that abnormally activated ERK signaling as a result of CSD may contribute to destruction of the TMJ by stimulating the production of MMPs.

Many studies have confirmed that psychological factors such as sleep disorders, psychological stress, and depression may relate to TMJ dysfunction [23]–[25]. Our study reinforces the idea that CSD in rats can indeed cause pathological changes in the TMJ at the molecular level.

MMPM is a well-established method that can effectively cause paradoxical sleep deprivation to rats without resulting in any additional stress such as social isolation or instability [26]–[28]. The grid on which the rats can lie down and sleep is considered as an adequate environmental control [5]. Many studies have demonstrated that paradoxical sleep deprivation may induce psychological stress and activate the hypothalamic-pituitary-adrenal (HPA) axis [29], [30]. Similar results were found in our study.

Our data showed that CSD could result in pathological and ultrastructural changes in the TMJ of rats. In our present experiment, histopathological changes, such as tough fibrous articular surfaces, fraction of distorted collagen fibers and debonding fibrous layer were observed in the CSD group, which were more serious than U0126 group at each time point. These findings confirmed that sleep deprivation may cause pathological alterations in rat TMJ and partly regulated by ERK pathway. So the ERK inhibitor, U0126, only extenuated the damages, but not reversed, the detailed mechanism would be taken in the further experiments. These findings reflect the correlation between sleep disorders and TMJ dysfunction. The HPA axis was activated and the rats exposed to sleep deprivation experienced a state of stress. Stressed rats have been demonstrated to exhibit obvious gnawing behavior and greater masseter muscle activity [31]–[33], both of which would exert more and greater jaw motion on the TMJ [34]. The imbalance between biosynthesis and degradation of matrix components may lead to TMJ synovitis and condylar cartilage destruction, both of which are important pathological features of TMD [35]. This is consistent with the findings of our previous study although the experimental rats were exposed to consecutive sleep deprivation [7]. In the previous study, we confirmed that, at the early stage, sleep deprivation could induce increases in the serum level of estradiol and synovitis, and intercellular edema in the synovial membrane of the TMJ [47], which were consistent with our previous findings [8]. These changes corresponded to the translocation of NF-κB p65 and the mRNA expression level of the inflammatory factors IL-1β, IL-6, TNF-α, and iNOS in the synovial membrane after sleep deprivation. At 5th and 7th day after sleep deprivation, the fluorescence signal of p65 in the nuclei of synovial cells significantly increased, which indicated that NF-κB pathway was activated [47]. Consistently, previous results showed that the mRNA expression of IL-1β, IL-6 and TNF-α significantly increased from 3rd day after SD and reached the peak expression at 7th day. Then the acute inflammatory reaction converted to a long-term inflammatory process, which plays a pivotal role in the development of TMD. The cytokine network plays an important role in TMJ inflammation. TNF-α, IL-1β, and IL-6 appear to be the major proinflammatory cytokines involved in TMJ pathology [9]. These cytokines can stimulate condylar chondrocytes proliferation and subsequent activation, which were involved in the pathologic process of inflammatory pain, and is associated with persistent inflammation and synovial membrane destruction in osteoarthritis [10].

To explore the molecular mechanism underlying pathological alterations in the TMJ following sleep deprivation, we examined the p-ERK, ERK, and MMP expression levels. In the MAPK pathway, ERK phosphorylation is a sign of ERK activation by MEK [10], [11]. U0126 is a chemically synthesized organic compound that inhibits the kinase activity of MEK [36]. It has been used in both in vivo and in vitro studies of MEK [37], [38]. The ERK pathway has been demonstrated to be a key factor in the induction of MMP-1, MMP-3, and MMP-13 in vitro [14], [15], [39]–[41]. These MMPs have been found to be upregulated in the synovial membrane and chondrocytes of TMJ diseases [42]–[44]. In our study, we found p-ERK activation and upregulation of MMP-1, MMP-3, and MMP-13 in the rats after they experienced sleep deprivation. When injected with U0126, p-ERK activation was blocked and the expression of these MMPs was downregulated, accompanied with remission of the pathological destruction of the TMJ. Together, these data indicate that the ERK signaling pathway is activated and the ERK phosphorylation level was significantly elevated following sleep deprivation, and that the increased downstream catabolic proteases and collagenases induced by ERK lead to pathological alterations of the synovial membrane and condylar cartilage in the TMJ. One interesting finding is that although the MMP-1, MMP-3, and MMP-13 expression levels showed obvious decreases after U0126 injection, the MMP-1 and MMP-13 levels in the U0126 group remained higher than those in the control group. A possible explanation for this finding could be that MMP synthesis depends on the simultaneous activation of many of the protein kinase pathways, including the c-Jun *N*-terminal kinase (JNK), ERK, p38, and WNT pathways, as reported previously [10], [45], [46]. Our previous study also demonstrated changes in the expression levels of c-fos and MAPK kinase 4 (MKK4), which are two key factors that act on the JNK signaling pathway [7]. Therefore, it is understandable that selective inhibition of a single protein kinase pathway induced only partial inhibition of MMP synthesis.

Most TMDs are self-limited with reparative effects and the majority of TMD patients do not exhibit any progressive damages to the TMJ. In our study, we did not observe any reparative effects upon histopathological examination of the rats of the experimental group that were exposed to sleep deprivation for up to 21 days. Therefore, we assume that repair of the TMJ following damage may require more time, and further studies are required to determine whether pathological alterations can be reversed if the experimental sleep deprivation is removed.

In summary, our study demonstrated that the histomorphology of the TMJ was altered by related molecular mechanisms caused when rats were exposed to sleep deprivation. These findings provide evidence for the possible involvement of sleep disturbances in the onset and progression of TMD. Therefore, sleep disturbances such as poor quality of sleep and sleep loss play important roles in TMD, indicating that such parameters should be taken into consideration in the treatment of TMJ disorders.
