# Correction: Effects of Chronic Sleep Deprivation on the Extracellular Signal-Regulated Kinase Pathway in the Temporomandibular Joint of Rats

**DOI:** 10.1371/journal.pone.0114988

**Published:** 2014-12-01

**Authors:** The PLOS ONE Staff

There is an error in the funding statement. Please refer to the correct funding statement below.

The authors received funding for this study from the following sources.

Grant number:Lu Cai Jiao Zhi (2013) 171;URL: http://www.sdcz.gov.cn/; Funding institution: Shandong Province Finance Bureau; Authors that received the funding: HZ;

Grant number:81400573; URL: http://www.nsfc.gov.cn/; Funding institution: National Natural Science Foundation of China; Authors that received the funding: WG;

Grant number:61471384; URL: http://www.nsfc.gov.cn/; Funding institution: National Natural Science Foundation of China; Authors that received the funding: WG.

## References

[1] Ma C, Wu G, Wang Z, Wang P, Wu L, et al (2014) Effects of Chronic Sleep Deprivation on the Extracellular Signal-Regulated Kinase Pathway in the Temporomandibular Joint of Rats. PLoS ONE 9(9): e107544 doi:10.1371/journal.pone.0107544 25226519 10.1371/journal.pone.0107544 PMC4167193

